# A Review of Health Risks and Pathways for Exposure to Wastewater Use in Agriculture

**DOI:** 10.1289/ehp.1509995

**Published:** 2016-01-29

**Authors:** Sarah K. Dickin, Corinne J. Schuster-Wallace, Manzoor Qadir, Katherine Pizzacalla

**Affiliations:** 1 School of Geography and Earth Sciences, McMaster University, Hamilton, Ontario, Canada; 2 United Nations University Institute for Water, Environment and Health, Hamilton, Ontario, Canada; 3 Sciences Po, Paris School of International Affairs, Paris, France

## Abstract

**Background:**

Wastewater is increasingly being used in the agricultural sector to cope with the depletion of freshwater resources as well as water stress linked to changing climate conditions. As wastewater irrigation expands, research focusing on the human health risks is critical because exposure to a range of contaminants must be weighed with the benefits to food security, nutrition and livelihoods.

**Objectives:**

The goal of this paper was to review research examining health risks and exposure pathways associated with wastewater irrigation to identify research trends and gaps.

**Methods:**

We conducted a review of the literature and identified a total of 126 studies published from 1995 to 2013. Findings were summarized based on several themes including types of exposure pathways, wastewater contaminants, methodological approaches and the geographical distribution of research.

**Results:**

Only 23 studies used epidemiological methods, while most research applied alternative methods to estimate risk, such as quantitative risk assessment models or comparisons of crop contamination to established guidelines for wastewater reuse. A geographic breakdown demonstrated a focus on microbiological contaminants in specific regions such as sub-Saharan Africa and Southeast Asia, despite growing chemical risks associated with rapid urbanization and industrialization that may change the types and distribution of wastewater contaminants.

**Conclusions:**

To provide a more comprehensive understanding of the health risks of wastewater use in agriculture, future research should consider multiple exposure routes, long-term health implications, and increase the range of contaminants studied, particularly in regions heavily dependent on wastewater irrigation.

**Citation:**

Dickin SK, Schuster-Wallace CJ, Qadir M, Pizzacalla K. 2016. A review of health risks and pathways for exposure to wastewater use in agriculture. Environ Health Perspect 124:900–909; http://dx.doi.org/10.1289/ehp.1509995

## Introduction

A growing demand for water to produce food, supply industries, and support human populations has led to competition for scarce freshwater supplies. Wastewater use is increasingly seen as an option to meet these growing needs for water. The agricultural sector is currently the largest user of water and wastewater globally, accounting for approximately 70% of water use on average (Winpenny et al. 2010). By contributing to food and water security, wastewater irrigation can alleviate strain on water resources by providing a reliable year-round source of water with sufficient nutrients for crop growth (Drechsel et al. 2010). This is especially critical in regions where climate change is expected to exacerbate water stress and increase precipitation variability (Hanjra et al. 2012). However, there are significant health implications associated with the use of wastewater for agriculture [World Health Organization (WHO) 2006].

### Wastewater Use in Agriculture—A Global Health Challenge

Wastewater is used for irrigation in treated and untreated forms, varying by geographic and economic context, albeit with the majority in untreated form in developing countries (Scott et al. 2010). Wastewater is commonly discharged into bodies of water with little or no treatment due to the limited availability of treatment facilities in many countries (Qadir et al. 2010). This untreated wastewater is frequently used for urban or peri-urban agriculture, which comprises approximately 11% of all irrigated croplands globally (Thebo et al. 2014). Untreated wastewater often contains a large range of contaminants from municipal, agricultural, and industrial sources. Excreta-related pathogens, skin irritants and toxic chemicals originating from these sources pose health risks to farmers and agricultural workers, their families, communities living in proximity to wastewater irrigation, as well as the consumers of wastewater-irrigated crops (Qadir et al. 2007). Wastewater exposure has been linked to viral, bacterial, and protozoan diseases such as salmonellosis, shigellosis, cholera, giardiasis, amoebiasis, hepatitis A, viral enteritis, and other diarrheal diseases (WHO 2006). In particular, helminth infections such as ascariasis are commonly associated with wastewater exposure, and are linked to anemia and impaired physical and cognitive development (Bos et al. 2010). Due to frequent contact with untreated wastewater, agricultural workers also experience skin diseases such as dermatitis and rashes. Exposure to heavy metals including arsenic, cadmium, lead, and mercury due to prolonged consumption of contaminated foods or occupational ingestion or inhalation of irrigated soil is linked to a wide range of chronic health effects. For instance, accumulation of cadmium, particularly in the kidneys, leads to kidney damage and osteoporosis. This is known as itai-itai disease in Japan, where the condition was originally linked to irrigation of rice paddies with highly contaminated water (Järup 2003). Emerging wastewater contaminants including polycyclic aromatic hydrocarbons (PAHs), personal care products, and endocrine-disrupting chemicals pose potential health risks; however, limited information is available on their uptake into food (Dodgen et al. 2013; Wang et al. 2012). Due to these widespread health risks, organizations including the WHO have developed guidelines to ensure that contaminant levels in wastewater are below limits that are harmful to human health (WHO 2006). However, since full treatment steps may not be possible, the WHO guidelines provide progressive targets for different wastewater situations.

While irrigation with untreated and inadequately treated wastewater presents a serious public health risk to farmers and consumers who are exposed to a range of contaminants, its use is important for smallholder livelihoods, particularly in economically disadvantaged regions or water-stressed areas (Raschid-Sally and Jayakody 2008). In many cities in developing regions, farmers in urban and peri-urban areas are dependent on wastewater to irrigate their crops and accept these risks despite significant contamination that contributes to a large burden of water-related disease (Qadir et al. 2010). The use of wastewater is also emerging as a form of climate change adaptation because it provides a consistent source of water in variable or dry conditions (Trinh et al. 2013). The complex drivers associated with wastewater use mean that steps to reduce health risks must be balanced with the need for increased food security, nutrition, and livelihoods (Drechsel et al. 2002). In the context of these health challenges the full extent of exposure to wastewater contaminants requires further exploration. The goal of this review was to critically examine the current literature (since the year 1995) reporting health risks and exposure pathways associated with wastewater use in agriculture.

## Methods

### Literature Search Strategy

Studies of potential interest were identified in three different databases, Web of Science, Science Direct, and PubMed using several different search phrases: “wastewater irrigation” and health; “wastewater irrigation” and disease; “wastewater irrigation” and safe; “wastewater reuse” and disease; “wastewater reuse” and health and crops; “wastewater reuse” and safe and crops; “irrigat* with reclaimed water” and crops; “greywater irrigation” and disease; “greywater irrigation” and health. Only papers that were peer reviewed, written in the English language, and published between the years 1995 and 2013 were included. There were no geographic restrictions placed on articles. In the case of several papers reporting the same study results, only the first paper was included.

After searching each database, individual article titles and abstracts were assessed to determine their relevance to the topic of this review. Three categories of empirical studies were included in the review: directly measured health risks of wastewater irrigation, indirectly measured health risks (e.g., using quantitative risk assessments or surveys), and studies that measured contamination of crops used for human consumption. Studies that solely measured soil or wastewater contamination levels were not included in the review. Other types of issues such as wastewater use in aquaculture and research focused only on soil health were also excluded.

### Data Extraction

To extract data from the included studies, the following information was collected from each article to ensure consistency:

Geographic focus and scale of research
Types of contaminants studied
Exposure pathways studied
Health risks identified
Information on contaminant uptake.

## Results

A total of 126 articles were identified for inclusion in the review. Only 23 studies applied epidemiological approaches to assess health risks directly by measuring prevalence of disease or chemical exposure in humans (Table 1). Of these, 15 studies assessed wastewater-related infections using stool samples and surveys, and in 4 studies skin ailments were assessed through dermatologist examination. For instance, using a nested case-control study of adults engaged in agricultural activities, Trang et al. (2007c) identified skin diseases including dermatitis and fungal infections associated with occupational exposure to untreated wastewater in Hanoi, Vietnam. Exposure was determined using interviews to collect information on contact with wastewater, personal hygiene practices, use of personal protective measures, and other relevant information such as contact with diseased persons and contact with animals. Gumbo et al. (2010) reported that farmers and their families using wastewater in Malamulele, South Africa, had a higher prevalence of helminth infections (42%) compared with control villages (27.5%), but depended on sales of vegetables grown with reliable wastewater supplies for their livelihoods. Srikanth and Naik (2004) found that the prevalence of giardiasis among farmers irrigating with wastewater in a suburb of Asmara, Eritrea, was 45%. Using hospital data on reported cases, they found that giardiasis prevalence was 7% among residents of the community who consumed only vegetables grown with untreated wastewater compared with 1% for residents in similar towns in Eritrea without wastewater irrigated crops. Melloul and Hassani (1999) found higher rates of *Salmonella* infection in children living in wastewater-irrigated areas near Marrakesh, Morocco, compared with those living in areas without wastewater irrigation. In addition to microbiological risks, three studies used blood or hair samples to test for heavy metal exposure (Chary et al. 2008; Cifuentes et al. 2000b; Lekouch et al. 1999). Not all studies identified risks from wastewater use. For example, Cifuentes et al. (2000b) found no association between agricultural work and Pb blood levels in the Mezquital Valley, Mexico, compared to other occupations. Habbari et al. (2000) found higher ascariasis prevalence in wastewater irrigation areas in Beni-Mellal, Morocco, but no association with trichuriasis prevalence compared to areas that did not practice wastewater irrigation. Devaux et al. (2001) found no health risks for farmers associated with spray irrigation using treated wastewater in Clermont-Ferrand, France, compared with non-exposed family members or farmers not using treated wastewater. While a few studies followed participants over time and were able to examine seasonal changes in health risks associated with farming activities (e.g., Trang et al. 2007d), studies largely used cross-sectional surveys to provide an understanding of risks.

**Table 1:** Summary of epidemiological studies that assessed health risks associated with wastewater irrigation.

Authors	Location	Data sources	Health risks	Contamination pathways considered	Study design
Amahmid and Bouhoum 2005	Marrakech Morocco	Stool samples, survey	Helminth infection (*Ascaris, Trichuris*)	Children living in wastewater irrigation areas, water, sanitation and hygiene, occupational (parents)	Cross-sectional study of an exposed group and control group children (*n* = 610)
Anh et al. 2007	Hanoi, Vietnam	Survey, dermatologist examination	Skin problems including infections, dermatitis and fungal growth	Occupational (farmers practicing aquatic plant culture)	Cross-sectional study with 2 follow-ups (*n* = 235 farmers) in 200 households in two communes, one using wastewater and another using river, rain, and well water
Anh et al. 2009	Phnom Penh, Cambodia	Survey, dermatologist examination	Skin problems including infections, dermatitis and fungal growth	Occupational (farmers practicing aquatic plant culture)	Cross-sectional study with 2 follow-ups (*n *= 650 adults in 200 households) in 5 villages (using different water sources)
Blumenthal et al. 2001	Mezquital Valley, Mexico	Stool samples, survey	Ascariasis and diarrhoeal disease	Occupational (workers and household), water, sanitation and hygiene, consumption of market vegetables	Cross-sectional survey with 850 agricultural households using untreated wastewater for irrigation, 950 households using wastewater stored in a reservoir, and 930 control households (rain-fed agriculture), with a total of 10,489 children and adults
Chary et al. 2008	Musi River, India	Samples of soils, vegetables, urine, blood, and livestock milk, survey	Irritation of skin with black rashes and other reactions possibly linked to heavy metal consumption (Zn, Cr, Cu, Ni, Co, and Pb)	Consumption of contaminated food (vegetables and milk)	Samples were collected from residents in the study region of varying ages and compared with control participants residing in the campus area
Cifuentes et al. 2000a	Mezquital Valley, Mexico	Stool samples, water samples, survey	Giardiasis	Occupational (workers and household), source of vegetables, water, sanitation and hygiene	Cross-sectional survey of farming households (children and adults). 2,257 individuals from an untreated wastewater group, 2,147 in a group using wastewater stored in a reservoir, and 2,344 in control group (rain-fed)
Cifuentes et al. 2000b	Mezquital Valley, Mexico	Blood samples, survey	Health impacts linked to Pb exposure	Occupational, use of Pb-glazed ceramics, crop consumption	Cross-sectional survey of 735 individuals (children and adults) from a farming population (households with at least one agricultural worker)
Devaux et al. 2001	Clermont-Ferrand, France	Sentinel reporting (physician/pharmacy), water and aerosol samples, health survey	Skin and digestive illnesses	Occupational (aerosol spraying), and accidental ingestion of those living in the area	Sentinel system from GPs and pharmacies, retrospective cohort of farmworkers (*n *= 100) and a prospective cohort of farmers using different water sources (*n *= 37), with 22 farmers using wastewater matched to non-exposed family members.
Ensink et al. 2005	Faisalabad, Pakistan	Stool samples, survey	Hookworm infection	Occupational (farmers and their children), water sanitation and hygiene, accidental ingestion of those living in the area, cattle ownership	Cross-sectional survey of wastewater farmers, textile labourers and farmers using regular irrigation water, with children assigned to their fathers’ exposure group (*n *= 1,704)
Ensink et al. 2006	Faisalabad, Pakistan	Stool samples, survey	Giardiasis	Occupational (farmers and their children), water sanitation and hygiene, accidental ingestion of those living in the area, cattle ownership	Cross-sectional survey of wastewater farmers, textile labourers and farmers using regular irrigation water, with children assigned to their fathers’ exposure group (*n *= 1,704)
Ensink et al. 2008	Hyderabad, India	Stool samples, survey, water samples	Intestinal nematode infection	Occupational (entire household), water, sanitation and hygiene, cattle ownership	Cross-sectional survey with 3 exposure groups of farmers exposed to untreated, and partially treated wastewater and river water (*n *= 1,078)
Gumbo et al. 2010	Malamulele, South Africa	Stool, vegetable and wastewater samples, survey	A range of parasitic infections including hookworm and *Giardia lamblia* infection	Occupational (entire household), consumption of contaminated vegetables, water, sanitation and hygiene	Cross-sectional survey of farmers and their children exposed to wastewater irrigation (*n *= 194) and those not using wastewater for irrigation (*n *= 249)
Habbari et al. 2000	Beni-Mellal, Morocco	Stool samples, survey	Helminth infection (*Ascaris, Trichuris*) in children	Children living near wastewater irrigated areas, water, sanitation and hygiene, occupation of parents	Cross-sectional survey of 740 children in communities using wastewater, and 603 children in communities not using wastewater irrigation
Hien et al. 2007	Hanoi, Vietnam	Stool samples, clinical information collected	Diarrhoeal diseases	Children of occupationally exposed farmers	Case–control study with 111 pairs of children
Lekouch et al. 1999	Marrakesh, Morocco	Hair samples, survey	Health risks linked to heavy metal (Pb and Cd) consumption (not specified)	Children living near irrigated areas, occupational exposure of parents, food	Cross-section survey of 327 children in a wastewater irrigation area, and 110 from control communities
Melloul and Hassani 1999	Marrakesh, Morocco	Stool sample, survey	*Salmonella* infection	Children living near irrigated areas, occupational exposure of parents	Cross-sectional survey of 390 children living a wastewater irrigation zone and 350 from control communities
Melloul et al. 2002	Marrakesh, Morocco	Stool sample, survey	*Salmonella* and protozoan infection	Children living near irrigated areas, occupational exposure of parents	Cross-sectional survey of children living in the wastewater irrigation zone and from control communities (*n *= 603 for *Salmonella* and *n *= 608 for protozoan infections)
Srikanth and Naik 2004	Asmara, Eritrea	Stool, wastewater and vegetable samples, survey, hospital data	Giardiasis and other gastrointestinal diseases	Occupational exposure (vegetable cultivation), consumption of vegetables, water, sanitation and hygiene	Cross-sectional health survey of 1,000 residents in farming community, stool samples collected from 75 occupationally exposed farmers
Trang et al. 2006	Nam Dinh city, Vietnam	Stool samples, wastewater survey	Helminth infection (*Ascaris, Trichuris* and hookworm)	Occupational (rice cultivation), water, sanitation and hygiene, use of human excreta for agriculture	Cross sectional survey, 202 households in a commune where wastewater was used for irrigation and 201 households in a commune that used river water, with a total of 1,088 individuals >15 years
Trang et al. 2007a	Hanoi, Vietnam	Stool samples, survey	Diarrhoeal diseases	Occupational (fish farming and cultivation of rice and vegetables) water, sanitation, and hygiene, use of human excreta for agriculture, animal husbandry	Open cohort of 636 adults engaged in agricultural work, and a nested case-control study with 163 unmatched pairs of cases and controls
Trang et al. 2007b	Hanoi, Vietnam	Stool samples, survey	Helminth infections (*Ascaris, Trichuris* and hookworm)	Occupational (workers and children) water, sanitation, and hygiene, use of human excreta for agriculture, animal husbandry	A cross-sectional study with 400 agricultural households (620 adults and 187 children)
Trang et al. 2007c	Hanoi, Vietnam	Dermatologist examination, survey	Skin ailments (itching often accompanied by skin chaps, debris or light ulcer; irritant contact dermatitis	Occupational (rice, aquatic and terrestrial vegetable cultivation), water, sanitation and hygiene, use of human excreta for agriculture, animal husbandry	Open cohort of 636 adults engaged in agricultural work and nested case–control study with 108 case control pairs
Trang et al. 2007d	Nam Dinh, Vietnam	Dermatologist examination, survey	Dermatitis, fungal infections, skin irritation (biological, inorganic and organic chemicals)	Occupational (e.g. rice and vegetable cultivation, flower growing) water, sanitation, and hygiene	Cohort of 400 households in two communes using wastewater and river water (1,103 individuals >15 years)

An additional 53 studies indirectly assessed health risks using methods such as surveys of perceived health impacts, quantitative microbial risk assessments (QMRA), and estimates of exposure by comparing crop consumption to the oral reference dose (e.g., health risk index) (see Table S1). Although risk modeling approaches such as QMRA have less financial limitations, these approaches are constrained by assumptions, such as estimates of accidental ingestion and the immune response of a population. The remaining 50 studies provided information on possible health risks by testing the level of contamination in crop samples and comparing this to established guidelines (e.g., WHO, U.S. Centers for Disease Control and Prevention, and European Union guidelines); however, this information is limited due to varying consumer exposure scenarios (see Table S2).

### Exposure Pathways

**Figure 1:**
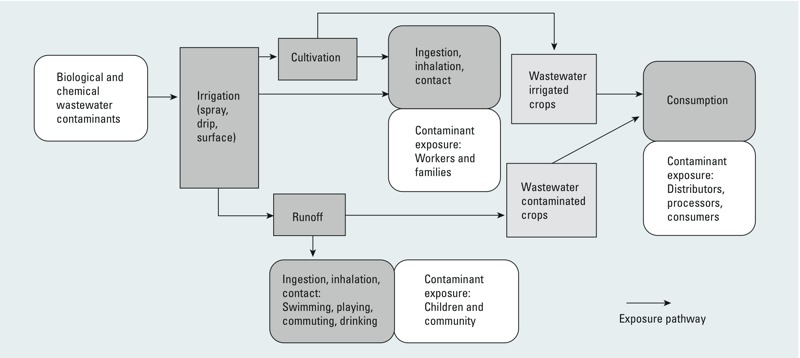
Exposure pathways associated with wastewater irrigation for consumers, agricultural workers and their families, and communities living in proximity.

A range of exposure pathways associated with wastewater irrigation was identified in the reviewed studies, illustrated in Figure 1 (Table 1; see also Tables S1 and S2). Consumption of crops was the most commonly studied contamination pathway, including both vegetables and cereals (*n* = 98). Research was primarily focused on foodstuffs, although one study examined contamination of crops used for medicinal products and supplements (Farahat and Linderholm 2013). In addition, Chary et al. (2008) and Yang et al. (2006) considered contamination of milk and meat, while several other studies examined fodder crops.

Farmworker exposure is significant because of the number of possible routes, the frequency and the duration of exposure. Many studies examined farmworker exposure (e.g., Forslund et al. 2010; Zhang et al. 2013), including several that also considered the impact on family members (e.g., An et al. 2007). Depending on the level of mechanization, this ranged from direct exposure to wastewater through planting and weeding (e.g., Trang et al. 2007b), variable exposure associated with different irrigation methods (e.g., gravity flow irrigation or manual irrigation with buckets) (Rutkowski et al. 2007), to accidental inhalation of aerosol sprays more common in wastewater use in high income countries (Carlander et al. 2009). Most research on farmworker exposure examined microbiological contaminants using epidemiological methods or QMRA, which were linked to health risks such as dermatitis, diarrhoea and helminth infections. Limited work was conducted on farmworker chemical exposure, although a few studies examined heavy metal intake (e.g., Cifuentes et al. 2000b; Yang et al. 2006). In the case of organic chemicals, a study performed in a wastewater irrigation area in China reported that the majority of estimated cancer risk from polycyclic aromatic hydrocarbon contamination of soil was attributable to exposure through soil ingestion and skin contact (i.e., worker exposure), while only 0.03% of the total estimated risk was attributed to exposure via crop consumption (Zhang et al. 2013).

In addition to occupational and food consumption risks, people living or commuting near wastewater-irrigated land may experience indirect exposure. For example, cricket games in proximity to contaminated soil were reported as possible sources of accidental ingestion (Ensink et al. 2006). Wastewater applied in aerosol form (i.e., sprays or sprinklers), was explored as irrigators and community members in the surrounding area can be exposed to pathogens through this irrigation method (Ayuso-Gabella et al. 2011; Carlander et al. 2009; Devaux et al. 2001). In high-income countries, there are still health risks for communities in proximity to wastewater irrigation areas where lower levels of treatment are used to grow non-food crops. For instance, contamination of groundwater and drinking water supplies, as well as nearby areas (e.g., fields with food crops) was linked to wastewater use for energy crops (used as sources of biomass) in Northern Ireland and Sweden (Carlander et al. 2009).

Children in particular are more vulnerable to some wastewater contaminants (Blumenthal et al. 2000), and this exposure was referenced in several articles (e.g., Mara et al. 2007; Yang et al. 2006). For instance, Blumenthal et al. (2001) found that children from agricultural households exposed to untreated wastewater had a much higher risk of *Ascaris lumbricoides* infection than those from the control group not irrigating with wastewater. Children may be exposed to wastewater through playing in contaminated areas, which increases the frequency and duration of environmental exposures (An et al. 2007). Children are also less likely to practice sanitary behaviors like hand washing (Nwachuku and Gerba 2004). In addition, children are often involved in helping with agricultural work. For instance, in a wastewater-irrigated area in Marrakesh, one study reported that boys who helped with agricultural work had a greater risk of Salmonella infection than girls who stayed at home (Melloul and Hassani 1999). However, all children in the wastewater area had a higher prevalence of disease compared with control areas, particularly those aged under 10.

**Figure 2:**
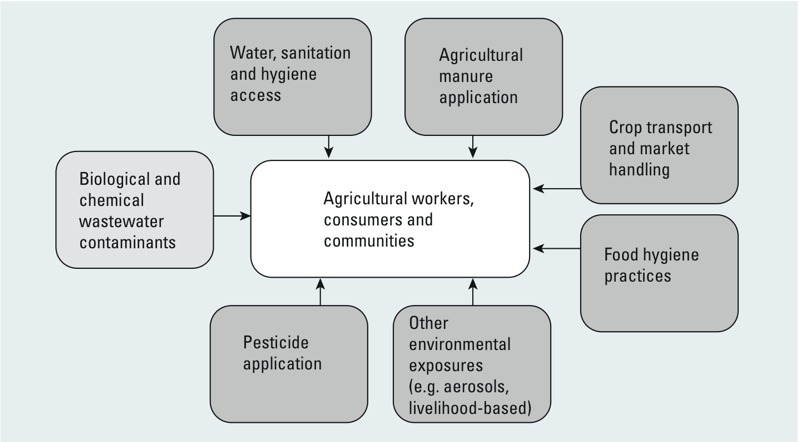
Wastewater exposure is one of multiple cumulative types of environmental exposure.

A key challenge to assessing the health risks of wastewater use is that people living in wastewater-irrigated areas may face exposures from a range of sources, and assessment of specific risk factors is made more difficult (Figure 2). In the reviewed studies this included poor living conditions, such as a lack of access to water for drinking, domestic uses and sanitation, which was considered in several studies (e.g., Blumenthal et al. 2001; Ensink et al. 2008). In addition, practices such as animal husbandry and use of excreta in agriculture were further pathways of exposure to excreta-related pathogens. For instance, Trang et al. (2007b) reported that wastewater use in peri-urban agriculture in Hanoi, Vietnam, did not increase the risk of helminth infection, while a lack of sanitation facilities and use of excreta for fertilizer did. Ferrer et al. (2012) identified a range of exposures to contaminants through recreational activities such as swimming, bathing, and fishing in canals contaminated with untreated domestic and industrial wastewater in Bangkok, in addition to exposures from vegetable growing. Increasing urbanization and industrial development in the Bangkok region have increased contamination levels in canals, so these multiple pathways of exposure present serious health risks.

Additionally, health risks have been identified due to mosquito vector breeding in wastewater that is treated in stabilization ponds until it is safe to use (Mukhtar et al. 2006). Agunwamba (2001) found that poorly maintained wastewater stabilization ponds in Nigeria created odor and mosquito issues, trapped livestock, and were associated with a greater burden of malaria among communities in proximity to the ponds, requiring improved maintenance and monitoring strategies to enhance the benefits of wastewater use.

While this review focused on farm-level contamination, additional contamination of crops may occur at various points along the production chain and a small number of studies compared market contamination to farm-level contamination. For example, in Faisalabad, Pakistan, Ensink et al. (2007) found that unhygienic market handling was the main source of microbiological contamination, including washing produce in wastewater drains, compared with farm-level wastewater irrigation.

### Contaminant Types and Methods

Contaminant types were divided into three groups comprising inorganic chemicals such as heavy metals, organic chemicals such as persistent organic pollutants, and microbiological contaminants including bacterial, viral, protozoan, and helminthic pathogens. Not including four studies that did not specify a type of contaminant (Anh et al. 2007, 2009; Dodgen et al. 2013; Friedler et al. 2006) or exposure to vectors (e.g., Mukhtar et al. 2006), the majority of studies focused on a single contaminant group (85%, *n* = 102), with only 2% conducted across all three contaminant groups (Downs et al. 1999; Jang et al. 2010; Trang et al. 2007d) (Table 1; see also Tables S1 and S2). Of the three types of wastewater contaminants, microbiological contaminants were considered in 53% of studies (*n* = 75), inorganic chemicals in 39% of studies (*n* = 55), and organic chemicals in 8% (*n* = 12) of studies. Work on organic contaminants included a large range of compounds including personal care products, polycyclic aromatic hydrocarbons (e.g., using petrochemical effluent to irrigate crops), and pesticides, primarily as skin irritants in Southeast Asia (e.g., Downs et al. 1999; Khan et al. 2008; Trang et al. 2007c).

Contaminant focus differed greatly by geographical region (Table 2). In general, studies in South Asia focused more on inorganic chemicals (e.g., Pb, Cd, Cr, and Zn). This corresponds to areas where industrial development has resulted in industrial effluent entering municipal wastewater and water bodies, presenting a risk of heavy metal exposure due to consumption of crops (Gupta et al. 2012; Jan et al. 2010; Tang et al. 2011). Studies in sub-Saharan Africa and Southeast Asia largely focused on microbiological contaminants, and chemical contaminants have generally been considered to be lower priority health risks in low-income countries (Bos et al. 2010). However, research is needed in areas experiencing growing industrial development in order to assess a greater number of contaminants (WHO 2006). This will be needed to identify, monitor and prioritize health risks, as well as to build capacity for enforcing safe disposal.

**Table 2:** Number of studies by contaminant type across several regions.

Contaminant type	Sub-Saharan Africa	South Asia and China	Southeast Asia	Middle East and North Africa	Latin America and the Caribbean	US, Europe, Australia
Microbiological	11	10	10	8	8	32
Inorganic chemicals (heavy metals)	7	28	2	5	2	9
Organic chemicals	1	4	1	0	1	2
Note: Some studies focus on more than one contaminant type. In addition, a few studies without a specific contaminant focus are not included in this count.						

Although there was little focus on organic chemical contamination, compounds such as endocrine disruptors, pharmaceuticals, PAHs, plasticizers, or flame retardants may pose risks to human health and environmental integrity. Regulatory guidelines for many of these contaminants are still being determined and many cannot be monitored or detected (Terzić et al. 2008). In general, these environmental contaminants are difficult and expensive to measure, despite representing a growing public health concern, especially in regions using treated wastewater that is considered safe (Dodgen et al. 2013). In developing countries there remains a larger contamination risk from pesticides applied directly to crops rather than from wastewater irrigation, and microbiological contaminants in wastewater continue to present a greater immediate health risk (Amoah et al. 2006; Simmons et al. 2010).

### Geographic Focus

Wastewater irrigation is practiced in different ways across many world regions and a geographic analysis provides important insight on the distribution of studies examining health risks (Table 1; see also Tables S1 and S2). The largest proportion of research was conducted in Asia, with 55 studies. The majority of these studies were undertaken in large wastewater irrigating regions in China, India and Pakistan, with only 13 studies conducted in Southeast Asia, including 9 located in Vietnam. Globally, these countries report some of the largest areas irrigated with untreated wastewater, with China the largest user of wastewater in absolute terms (Jiménez and Asano 2008).

The Middle East and North Africa (MENA) region report the largest number of water-stressed countries and highest water withdrawals for agriculture of any region in the world. Seven countries in the MENA region are within the top 10 highest wastewater users per capita (Jiménez and Asano 2008). Despite intensive wastewater use in the region, only 14 of the reviewed articles had this geographic focus, with 6 focused on Morocco. More research is needed on health risks of wastewater use in these water-stressed areas relying heavily on such waters for irrigation (Grangier et al. 2012).

The sub-Saharan Africa region, where most wastewater used for irrigation is untreated, was the focus of 18 studies, including 4 studies located in Ghana (Amoah et al. 2006; Keraita et al. 2008; Lente et al. 2012; Silverman et al. 2013). The majority of these studies focused on urban vegetable farming using untreated wastewater discharged into urban drainage systems. Latin America and the Caribbean, where only about 20% of generated wastewater is treated (Sato et al. 2013), was the focus of only 11 studies despite high levels of wastewater use, 8 of which examined wastewater use in Mexico. For instance we found no studies conducted in Chile, a country which irrigates the most hectares with treated wastewater in the world (Jiménez and Asano 2008).

In the European region, 27 study sites were identified with the largest number in Spain and Italy, while 8 studies were identified in Australia and the United States. The majority of these studies used experimental plots or were greenhouse studies (i.e., controlled plots testing various crop or treatment variables), with exceptions that examined the use of wastewater for crops such as olive trees or biofuel (e.g., Palese et al. 2009; Pedrero et al. 2012). Overall, these findings show that research is concentrated in several regions and countries, while other regions where wastewater irrigation is extensively practiced remain under-researched.

### Contaminant Uptake

Contaminant uptake into the food chain is a key concern associated with wastewater irrigation, as this is the most widespread exposure route. Accumulation of even small amounts of metals in soil is a challenge in areas with long-term wastewater irrigation due to chronic exposure for consumers. In the case of heavy metals and metalloids, uptake is determined by a range of factors including soil conditions and vegetable type. For instance, Khan et al. (2008) found that leafy vegetables had higher transfer of Cd, Cu, and Ni from soil to plants in Tianjin, China. Singh et al. (2010) compared a range of vegetables contaminated with heavy metals near Varanasi, India and found highest concentrations in cabbage, brinjal (eggplant) and the leafy vegetables lady’s ﬁnger and spinach, compared with other vegetables such as gourd varieties. However, although heavy metal concentration in rice and wheat were lower in this analysis, they were found to be a greater risk to people than other crops due to their higher dietary consumption levels. Uptake of organic contaminants has also been found to vary by vegetable type (Calderón-Preciado et al. 2013). Dodgen et al. (2013) found that the charge of organic compounds impacted plant uptake when comparing collards and lettuce. Wu et al. (2013) identified organic compounds that preferentially sorbed to roots versus those that easily translocated into leaves, but noted that estimation of dietary intake by humans implied negligible risks.

Information on varying uptake levels among crop types might be used to determine which vegetables should be grown in areas with specific contaminants (Simmons et al. 2010). For instance, in a comparison of leafy vegetables in Nigeria, Agbenin et al. (2008) found lettuce contained much higher levels of As and Cr, but lower levels of Cd compared with amaranth. Wang et al. (2003) found that wheat accumulated more Cd and Pb in the seeds than corn, where the metals accumulated in the roots. However, many context-specific factors must be considered when making crop recommendations and not all are based on consumer health, such as market value and growing conditions.

In addition to uptake of chemicals through roots, contaminants can be retained on the surface of crops and vegetables that grow above ground. Hamilton et al. (2006) explored the potential impact of pathogen retention using a QMRA model projecting enteric virus survival on leafy vegetables (lettuce, cabbage and broccoli) and cucumber. The smoother cucumber skin retained less water and therefore lower virus amounts, while cabbage retained more water and greater virus amounts than broccoli. Pandey et al. (2012) found that while root vegetables showed greater uptake of heavy metals over leaves and fruits irrigated with wastewater, when the inputs of atmospheric deposition were considered together with wastewater irrigation, leafy vegetables showed greater contamination levels. A challenge for understanding and comparing risks identified in these studies is the range of methods used to assess contamination, including dry or wet weight of chemicals and ratios of soil to plant matter.

## Implications

Wastewater irrigation is expected to grow in countries with freshwater scarcity, especially in light of increasing volumes of urban wastewater (Sato et al. 2013). This requires an in-depth understanding of the major health risks and exposure pathways in order to make relevant risk management decisions for different wastewater use situations. Several recommendations and areas for additional research on wastewater health risks emerge from our results.

### Recognition of Complex Pathways

Research is needed to broaden understanding of complex risk factors associated with wastewater use, such as evaluating multiple types of contaminant groups, cumulative exposure pathways in wastewater irrigation areas, and inconsistent contaminant concentrations in wastewater. The most common exposure pathway analyzed was consumption of wastewater-irrigated crops, however researchers must also consider synergies with other environmental health risks when designing guidelines. For example, Pandey et al. (2012) showed that atmospheric deposition of toxic metals was an additional source of metal contamination in vegetables near Varanasi, India. Similarly, Lente et al. (2012) suggested that although Pb levels in vegetables grown in Accra, Ghana, were found to be below dietary thresholds [based on estimated daily intake compared with FAO/WHO (2010) and WHO (2006) thresholds], there were additional routes of Pb exposure in the environment. The importance of other compounding exposures such as water and sanitation access, use of excreta as fertilizer, and animal husbandry, among farmers, consumers and those living in wastewater irrigation areas must be understood in order to best target resources for protecting and improving health.

In the case of crop contamination, transport of contaminants in the environment and variable uptake into crops, as well as differing types of food consumed should be considered. A risk assessment by Hamilton et al. (2006) suggested that the risk of enteric infection due to infrequent consumption of highly contaminated vegetables was comparable to the estimated risk due to more frequent consumption of crops with far lower contamination levels. While thoroughly washing and preparing food prior to consumption can minimize or eliminate microbiological risks and reduce some chemical risks, uptake of chemical contaminants by plants is harder to address. In some areas where wastewater irrigation has been applied for decades (e.g., Tai et al. 2013), contaminant bioaccumulation in soil has reached dangerous levels, especially in cases where consumers have lifelong exposure to contaminants. However, chronic health outcomes among consumers, such as cancers, are more difficult to attribute to wastewater (Bos et al. 2010; Gupta et al. 2012), and more long-term studies are needed to increase understanding of these health risks.

Agricultural workers and their families face immediate health risks from direct contact with contaminated soil (e.g., through ingestion and inhalation) (An et al. 2007), and fewer studies analyzed these risks compared with crop contamination studies. Measuring occupational health risks for agricultural workers is challenging because of varying time lengths of exposure, and changing contaminant concentration due to seasonal differences in water availability or treatment level of effluent used for irrigation. For instance, Rutkowski et al. (2007) reported that in the Kathmandu Valley wastewater irrigation was used in the dry season when pollutant levels were highest in order to grow vegetable cash crops in water scarce conditions. In addition, different farming practices and irrigation techniques may impact exposure to wastewater, and duties may differ based on gender roles. More work is needed where the risks to farmers are especially high in the context of informal urban agriculture, where barriers such as insecure land tenure and lack of resources reduce investment in health protection measures such as drip irrigation and protective clothing (Drechsel et al. 2002). This may be due to the fact that untreated wastewater irrigation remains in the informal sector in most developing countries. As such, wastewater irrigation is not often acknowledged at the national level due to the fear of economic repercussions in agricultural trade, requiring food safety and other phyto-sanitary measures (Qadir et al. 2010). In addition, attention to vulnerable groups is needed, such as children involved in agricultural work who face disproportionate risks.

In addition to agricultural wastewater contamination, food contamination can occur at other points in the food supply and processing chain from sources such as transportation, market selling or household food hygiene (WHO 2015). Greater consideration of additional contamination routes can an improve targeted risk reduction measures that focus on the greatest hazards, while still supporting livelihoods of farmers and ensuring a secure urban food supply. For instance, Amoah et al. (2006) found harmful levels of microbial and pesticide contamination on produce sold in Ghanaian markets. They suggested this may due to irrigation water quality as well as market washing practices, and recommended education and awareness campaigns on food hygiene for markets and households to protect public health without limiting farmers’ livelihoods.

There are additionally research opportunities to provide a better understanding of the ‘multiple realties’ wastewater use, including benefits such as water and food security, nutrient input and improved livelihoods, which should be considered together with the risks (Raschid-Sally 2013). For instance, Pedrero et al. (2012) found that fruit-quality index values (e.g., color, weight, peel thickness) were significantly better in fruit irrigated with secondary treated wastewater, compared with tertiary treated wastewater (a more advanced treatment). Moreover, children in wastewater irrigation areas in Vietnam were found to have better nutritional status than those in areas using river water for irrigation (Trang et al. 2006). Thus, recommendations to reduce risk must be based on local situations, such as reducing celery cultivation where it indicates greatest uptake potential of Cd (Tang et al. 2011) or changing irrigation methods for lettuce growing in areas where it risks retaining pathogens that are protected from sunlight and desiccation. However, recommendations to reduce exposure to contaminants may not be acceptable or economically feasible to some populations who are accustomed to eating or selling certain vegetables. In addition, farmers prefer to grow certain high-value vegetables for markets and the year-round availability of wastewater irrigation can increase profit margins (Qadir et al. 2000). The standardization of research methods, such as consistent methodologies and reporting relating to contaminant uptake into crops could improve guidelines to ensure more effective risk management.

### Changing Urban Environments

Research on wastewater in low and middle income countries with expanding urban populations and rapidly growing industrial sectors must reflect these changing conditions. For example, few studies addressed more than one type of contamination, which may provide an incomplete picture of health risks in these contexts. While urban farming activities in sub-Saharan Africa and Southeast Asia have traditionally faced a greater risk of biological contamination, more evidence is needed in areas where new exposures to chemical contaminants threaten public health. In addition, transport and uptake of emerging organic compounds in the environment, and their health risks, are poorly understood yet increasingly relevant to developed countries using treated wastewater where these compounds may not be removed during conventional treatment (Daughton 2004). Furthermore, rather than an even distribution of knowledge, existing research was focused in specific geographical regions. Research is needed in a greater number of regions where wastewater irrigation is applied, as seven countries accounted for 50% of reviewed studies (China, India, Mexico, Pakistan, Spain, Vietnam, USA). Of these countries, China and Mexico irrigate by far the greatest number hectares with untreated wastewater, but represent only around 10% of general wastewater reuse per capita (Jiménez and Asano 2008).

Better testing and monitoring methods will be required to ensure that the full range of health risks is considered by decision-makers, particularly with increasing global food trade. In a global assessment, Sato et al. (2013) reported limited availability of information on use of wastewater in many countries, however the human health risks are a critical reason to motivate collection of this information. Although the majority of studies in this review applied indirect risk assessment approaches (e.g., QMRA or health risk index) or assessment of crop contamination, more epidemiological research is required that directly tests disease prevalence and exposures to chemical contaminants in order to provide a stronger evidence-base for decision-makers. In addition to changing social and economic conditions and priorities, research on the impact of climate change on wastewater use will be valuable. This will improve understanding of the advantages for adaptation in water-stressed areas, as well as the risks for increased survival of pathogens such as *Salmonella* in warmer temperatures (Schuster-Wallace et al. 2014).

### Knowledge, Attitudes, and Costs

More research to characterize knowledge, attitudes and behaviors relating to wastewater health risks for farmers and consumers in different geographical regions will provide a greater understanding of the extent to which guidelines are observed. For instance, in some cases the economic benefits of using wastewater and the reduced need for fertilizers are the primary incentives for farmers to use wastewater irrigation (Amerasinghe et al. 2009). Understanding diverse perspectives on wastewater use can help identify useful awareness building and education activities to mitigate health risks. While hygienic practices and protective equipment use can be implemented to reduce risks, they can be prohibitively expensive, e.g., for migrant workers, and may need to be monitored and enforced or incentivized to be effective. In addition, understanding consumer practices relating to hygienic cooking practices including washing vegetables with clean water is critical. These types of research opportunities can promote collaboration across social and physical sciences in order to incorporate different forms of evidence to develop improved and comprehensive recommendations. Finally, economic evaluations and analyses of the acute and chronic healthcare costs compared with benefits of wastewater use, such as increases in nutrition and food security, can provide further useful evidence for decision makers.

## Conclusions

As the use of wastewater in agriculture expands to respond to freshwater scarcity, it is critical to remain informed of health risks associated with the practice. In areas traditionally dealing with microbiological contaminants, such as Southeast Asia and sub-Saharan Africa, there is a need to assess a growing number of chemical contaminants produced by emerging industrial sectors and increasingly concentrated wastewater associated with large urban populations. In developed regions, where wastewater is treated for standard contaminants such as pathogens and inorganic chemicals, organic contaminants are an area for further investigation. Work in under-researched regions, such as Latin America, would enhance evidence available for decision-making in these areas.

Developing wastewater guidelines that achieve a balance between promoting health and protecting other benefits, including farmer livelihoods and a secure food supply, is challenging and depends on adequate health data. Despite serious health risks, we found few studies that directly assessed disease prevalence or chemical exposure associated with wastewater use, compared with a much larger body of work using risk assessment models (e.g., QMRA) or crop samples to estimate risks. While these studies require greater resources, the information that they provide is necessary to support context-specific guidelines and decision-making and is currently limited. Moreover, studies focused on long-term exposure are needed to analyze the risks of ongoing occupational or food-based exposure to low levels of contamination. Finally, multiple cumulative environmental exposures affecting populations in wastewater use areas, such as water and sanitation access, must be comprehensively examined to prioritize risk reduction steps and to target vulnerable groups. The research gaps identified in this review provide insight on opportunities for future work that will contribute to harnessing the benefits of wastewater in changing environmental and social contexts, while mitigating risks to public health.

## Supplemental Material

(169 KB) PDF
