# Supplementary material for: A Review of Health Risks and Pathways for Exposure to Wastewater Use in Agriculture
Source: Environ Health Perspect. 2016 Jan 29;124(7):900–9. doi: 10.1289/ehp.1509995 (PMC4937861; doi:10.1289/ehp.1509995)
Supplement: (169 KB) PDF [file ehp.1509995.s001.acco.pdf]

Environ Health Perspect
DOI: 10.1289/ehp.1509995

Note to readers with disabilities: *EHP* strives to ensure that all journal content is accessible to all readers. However, some figures and Supplemental Material published in *EHP* articles may not conform to 508 standards due to the complexity of the information being presented. If you need assistance accessing journal content, please contact ehp508@niehs.nih.gov. Our staff will work with you to assess and meet your accessibility needs within 3 working days.

Supplemental Material

A Review of Health Risks and Pathways for Exposure to Wastewater Use in Agriculture

Sarah K. Dickin, Corinne J. Schuster-Wallace, Manzoor Qadir, and Katherine Pizzacalla

Table of Contents

Table S1. Description of studies using indirect methods to assess health risks

Table S2. Description of crop contamination studies

Table S1. Description of studies using indirect methods to assess health risks

	Citation	Region	Country	Types of exposure[a]	Type of exposure pathway[b]
1	Agunwamba, 2001	Africa	Nigeria	Biological, Vector-borne	1,2,9
2	Aiello et al., 2013	Europe	Italy	Biological	1
3	An et al., 2007	Asia	Korea	Biological	2, 4
4	Ayuso-Gabella et al., 2011	Australia, MENA, Europe	Australia, Israel, Spain, Italy	Biological	1,2,5
5	Barker et al., 2013	Australia	Australia	Biological	1,2,9
6	Bastos et al., 2008	Latin America	Brazil	Biological	1
7	Carlander et al., 2009	Europe	Greece, Sweden, northern Ireland	Biological	2,4,5,8, 10, 11
8	Diallo et al., 2008	Asia	Thailand	Biological	1, 2, 4, 11
9	Downs et al., 1999	Latin America	Mexico	Inorganic, biological, organic	10
10	Ferrer et al., 2012	Asia	Thailand	Biological	1,2, 11
11	Forslund et al., 2010	Europe	Serbia, Italy	Biological	1,2
12	Forslund et al., 2012	Europe	Crete, Italy	Biological	1,2
13	Friedler et al., 2006	MENA	Syria	No focus	
14	Grangier et al., 2012	MENA	Israel	Biological	4
15	Gupta et al., 2012	Asia	India	Inorganic	1
16	Hamilton et al., 2006	USA and Canada	USA	Biological	1
17	Jan et al., 2010	Asia	Pakistan	Inorganic	1
18	Jang et al., 2010	Asia	Korea	Inorganic, biological, organic	1,2
19	Keraita et al., 2008	Africa	Ghana	Biological	1,2
20	Khan et al., 2013	Asia	Pakistan	Inorganic	1
21	Khan et al., 2008	Asia	China	Inorganic	1
22	Lente et al., 2012	Africa	Ghana	Inorganic	1
23	Lim and Jiang, 2013	USA and Canada	USA	Biological	1
24	Mahmood and Malik, 2013	Asia	Pakistan	Inorganic	1
25	Mapanda et al., 2007	Africa	Zimbabwe	Inorganic	1
26	Mara et al., 2007	Latin America	Mexico	Biological	1,2,4
27	Mojid et al., 2010	Asia	Bangladesh	Inorganic and biological	2
28	Moradmand and Harchegani, 2011	MENA	Iran	Inorganic	1
29	Mukhtar et al., 2006	Asia	Pakistan	Vector-borne	9
30	Mukhtar et al., 2003	Asia	Pakistan	Vector-borne	9
31	Munoz et al., 2010	Europe	Spain	Biological, organic	1
32	Mutengu et al., 2007	Africa	Zimbabwe	Inorganic and biological	1,2
33	Nabulo et al., 2010	Africa	Uganda	Inorganic	1
34	Navarro and Jimenez, 2011	Latin America	Mexico	Biological	1
35	Pandey et al., 2012	Asia	India	Inorganic	1
36	Petterson and Ashbolt, 2001	USA and Canada	USA	Biological	1

37	Petterson et al., 2001	None	None	Biological	1
38	Ramadan and Mandil, 2009	MENA	Syria	Inorganic	1
39	Rutkowski et al., 2007	Asia	Nepal	Biological	2
40	Shuval et al., 1997	None	None	Biological	1
41	Silverman et al., 2013	Africa	Ghana	Biological	
42	Singh et al., 2010	Asia	India	Inorganic	1
43	Srinivasan and Reddy, 2009	Asia	India	Inorganic, biological	1,2
44	Tang et al., 2011	Asia	China	Inorganic	1
45	Wang et al., 2013 a	Asia	China	Inorganic	1
46	Wang et al., 2012a	AsiaLatin America	China	Organic	1
47	Wang et al., 2013b	Asia	China	Inorganic	1
48	Wang et al., 2012b	Asia	China	Inorganic	1
49	Wang et al., 2012c	Asia	China	Inorganic	1
50	Weldesilassie et al., 2011	Africa	Ethiopia	Biological	1,2,7
51	Wu et al., 2013	None	None	Organic	1
52	Yang et al., 2006	Asia	China	Inorganic	1,2,4,6
53	Zhang et al., 2013	Asia	China	Organic	1,2,5,8

[a]Contaminant types were divided into three groups comprising inorganic chemicals such as heavy metals, organic chemicals such as persistent organic pollutants, plasticizers and pharmaceutics, and microbiological contaminants including bacterial, viral, protozoan, and helminthic pathogens. Exposure to vector-borne disease, associated with mosquito breeding in wastewater is also noted. A few studies that mention no specific focus, such as 'wastewater contaminants' are noted as such.

[b]Codes for exposure pathways: 1 = Consumed crops; 2 = Farmer occupational; 3 = Farmer's family occupational; 4 = Children playing in irrigated area; 5 = Aerosols or inhalation of soil particles; 6 = Milk/mean from irrigated fodder; 7 = Water, sanitation, hygiene; 8 = People living in contaminated area, e.g. commuting, recreation; 9 = Vectors breeding in wastewater; 10 = Consumption of contaminated groundwater; 11 = Contact with surface water/swimming; 12 = Animal husbandry/contact with animals

Table S2. Description of crop contamination studies

	Citation	Region	Country	Types of exposure[a]	Type of exposure pathway[b]
1	Abdu et al., 2011	Africa	Nigeria, Burkina Faso and Mali	Inorganic	1
2	Achakzai and Bazai, 2006	Asia	Pakistan	Inorganic	1
3	Agbenin et al., 2009	Africa	Nigeria	Inorganic	1
4	Ahmad and Goni, 2010	Asia	Bangladesh	Inorganic	1
5	Akponikpè et al., 2011	Africa	Burkina Faso	Biological	1
6	Al-Sa'ed, 2007	MENA	Palestine	Biological	1, 4, 8
7	Amoah et al., 2006	Africa	Ghana	Biological and organic	1
8	Anh et al., 2007a	Asia	Cambodia	Biological	1
9	Arora et al., 2008	Asia	India	Inorganic	1
10	Assadian et al., 2005	USA and Canada	USA	Biological	1
11	Assadian et al., 1998	USA and Canada, Latin America	USA, Mexico	Inorganic	1
12	Avci, 2012	Europe	Turkey	Inorganic	1
13	Avci and Deveci, 2013	Europe	Turkey	Inorganic	1
14	Aziz et al., 1996	Asia	India	Inorganic and organic	1
15	Bastos and Mara, 1995	Europe	Portugal, England	Biological	1
16	Bichai et al., 2012	Europe	Spain	Biological	1
17	Calderon-Preciado et al., 2013	Europe	Spain	Organic	1
18	Castro et al., 2013	Europe	Spain	Biological and inorganic	1
19	Choi et al., 2004	USA and Canada	USA	Biological	1
20	Dodgen et al., 2013	No focus	No focus	No focus	1
21	Duan et al., 2010	Asia	Pakistan	Inorganic	1
22	Ensink et al., 2007	Asia	China	Biological	1
23	Farahat and Linderholm, 2013	MENA	Egypt	Inorganic	1,6
24	Fasciolo et al., 2002	Latin America	Argentina	Biological	1
25	Gemmell and Schmidt, 2012	Africa	South Africa	Biological	1
26	Gupta et al., 2008	Asia	India	Inorganic	1
27	Gupta et al., 2009	Asia	India	Biological	1,2
28	Kalavrouziotis et al., 2008	Europe	Greece	Inorganic and biological	1
29	Kang et al., 2007	Asia	Korea	Inorganic	1
30	Khai et al., 2007	Asia	Vietnam	Inorganic	1
31	Sardar Khan et al., 2008	Asia	China	Inorganic and organic	1
32	Manas et al., 2009	Europe	Spain	Inorganic and biological	1
33	Manios et al., 2006	Europe	Greece	Biological	1
34	Melloul et al., 2001	MENA	Morocco	Biological	1,2
35	Mendoza-Espinosa et al., 2008	Latin America	Mexico	Biological	1

36	Minhas et al., 2006	Asia	India	Biological	1
37	Mojid and Wyseure, 2013	Asia	Bangladesh	Inorganic and biological	1
38	Njenga et al., 2011	Africa	Kenya	Inorganic and biological	1
39	Palacios et al., 2001	Europe	Spain	Biological	6
40	Palese et al., 2009	Europe	Italy	Biological	1
41	Papadopoulos et al., 2009	Europe	Greece	Inorganic and biological	1
42	Pedrero and Alarcón, 2009	Europe	Spain	Inorganic and biological	1
43	Pedrero et al., 2012	Europe	Spain	Inorganic and biological	1
44	Peters and Ngai, 2004	Asia	Vietnam	Biological	1,2
45	Platzer et al., 2004	Latin America	Nicaragua	Biological	1,2
46	Qadir et al., 2000	Asia	Pakistan	Inorganic	1
47	Rusan et al., 2007	MENA	Jordan	Inorganic	6
48	Sharma et al., 2007	Asia	India	Inorganic	1
49	Tai et al., 2013	Asia	China	Inorganic	1
50	Wang et al., 2003	Asia	China	Inorganic	1

[a]Contaminant types were divided into three groups comprising inorganic chemicals such as heavy metals, organic chemicals such as persistent organic pollutants, plasticizers and pharmaceutics, and microbiological contaminants including bacterial, viral, protozoan, and helminthic pathogens. Exposure to vector-borne disease, associated with mosquito breeding in wastewater is also noted. A few studies that mention no specific focus, such as 'wastewater contaminants' are noted as such.

[b]Codes for exposure pathways: 1 = Consumed crops; 2 = Farmer occupational; 3 = Farmer's family occupational; 4 = Children playing in irrigated area; 5 = Aerosols or inhalation of soil particles; 6 = Milk/mean from irrigated fodder; 7 = Water, sanitation, hygiene; 8 = People living in contaminated area, e.g. commuting, recreation; 9 = Vectors breeding in wastewater; 10 = Consumption of contaminated groundwater; 11 = Contact with surface water/swimming; 12 = Animal husbandry/contact with animals.

References

Abdu N, Abdulkadir A, Agbenin JO, Buerkert A. 2011. Vertical distribution of heavy metals in wastewater-irrigated vegetable garden soils of three West African cities. Nutr. Cycl. Agroecosystems 89:387–397; doi:10.1007/s10705-010-9403-3.

Achakzai AKK, Bazai ZA. 2006. Phytoaccumulation of heavy metals in spinach (Spinacea oleraceac L.) irrigated with wastewater of Quetta City. J. Chem. Soc. Pakistan 28: 473–477.

Agbenin JO, Danko M, Welp G. 2009. Soil and vegetable compositional relationships of eight potentially toxic metals in urban garden fields from northern Nigeria. J. Sci. Food Agric. 89:49–54; doi:10.1002/jsfa.3409.

Agunwamba JC. 2001. Analysis of socioeconomic and environmental impacts of waste stabilization pond and unrestricted wastewater irrigation: Interface with maintenance. Environ. Manage. 27:463–476; doi:10.1007/s002670010162.

Ahmad JU, Goni MA. 2010. Heavy metal contamination in water, soil, and vegetables of the industrial areas in Dhaka, Bangladesh. Environ. Monit. Assess. 166:347–357; doi:10.1007/s10661-009-1006-6.

Aiello R, Cirelli GL, Consoli S, Licciardello F, Toscano A. 2013. Risk assessment of treated municipal wastewater reuse in Sicily. Water Sci. Technol. 67:89–98; doi:10.2166/wst.2012.535.

Akponikpè PBI, Wima K, Yacouba H, Mermoud A. 2011. Reuse of domestic wastewater treated in macrophyte ponds to irrigate tomato and eggplant in semi-arid West-Africa: Benefits and risks. Agric. Water Manag. 98:834–840; doi:http://dx.doi.org/10.1016/j.agwat.2010.12.009.

Al-Sa’ed R. 2007. Pathogens assessment in reclaimed effluent used for industrial crops irrigation. Int J Env. Res Public Heal. 4: 68–75.

Amoah P, Drechsel P, Abaidoo RC, Ntow WJ. 2006. Pesticide and pathogen contamination of vegetables in Ghana’s urban markets. Arch. Environ. Contam. Toxicol. 50:1–6; doi:10.1007/s00244-004-0054-8.

An YJ, Yoon CG, Jung KW, Ham JH. 2007. Estimating the microbial risk of E-coli in reclaimed wastewater irrigation on paddy field. Environ. Monit. Assess. 129:53–60; doi:10.1007/s10661-006-9425-0.

Anh VT, Tram NT, Klank LT, Cam PD, Dalsgaard A. 2007. Faecal and protozoan parasite contamination of water spinach (Ipomoea aquatica) cultivated in urban wastewater in Phnom Penh, Cambodia. Trop. Med. Int. Heal. 12:73–81; doi:10.1111/j.1365-3156.2007.01944.x.

Arora M, Kiran B, Rani S, Rani A, Kaur B, Mittal N. 2008. Heavy metal accumulation in vegetables irrigated with water from different sources. Food Chem. 111:811–815; doi:10.1016/j.foodchem.2008.04.049.

Assadian NW, Di Giovanni GD, Enciso J, Iglesias J, Lindemann W. 2005. The transport of waterborne solutes and bacteriophage in soil subirrigated with a wastewater blend. Agric. Ecosyst. Environ. 111:279–291; doi:http://dx.doi.org/10.1016/j.agee.2005.05.010.

Assadian NW, Esparza LC, Fenn LB, Ali AS, Miyamoto S, Figueroa U V, et al. 1998. Spatial variability of heavy metals in irrigated alfalfa fields in the upper Rio Grande River basin. Agric. Water Manag. 36:141–156; doi:http://dx.doi.org/10.1016/S0378-3774(97)00054-1.

Avci H. 2012. Trace metals in vegetables grown with municipal and industrial wastewaters. Toxicol. Environ. Chem. 94:1125–1143; doi:10.1080/02772248.2012.691501.

Avci H, Deveci T. 2013. Assessment of trace element concentrations in soil and plants from cropland irrigated with wastewater. Ecotoxicol. Environ. Saf. 98:283–291; doi:10.1016/j.ecoenv.2013.08.013.

Ayuso-Gabella N, Page D, Masciopinto C, Aharoni A, Salgot M, Wintgens T. 2011. Quantifying the effect of Managed Aquifer Recharge on the microbiological human health risks of irrigating crops with recycled water. Agric. Water Manag. 99:93–102; doi:http://dx.doi.org/10.1016/j.agwat.2011.07.014.

Aziz O, Inam A, Samiullah, Siddiqi RH. 1996. Long term effects of irrigation with petrochemical industry wastewater. J. Environ. Sci. Heal. Part a-Environmental Sci. Eng. Toxic Hazard. Subst. Control 31: 2595–2620.

Barker SF, O'Toole J, Sinclair MI, Leder K, Malawaraarachchi M, Hamilton AJ. 2013. A probabilistic model of norovirus disease burden associated with greywater irrigation of home-produced lettuce in Melbourne, Australia. Water Res. 47:1421–1432; doi:10.1016/j.watres.2012.12.012.

Bastos RK, Bevilacqua PD, Silva CA, Silva C V. 2008. Wastewater irrigation of salad crops: further evidence for the evaluation of the WHO guidelines. Water Sci Technol 57:1213–1219; doi:10.2166/wst.2008.244.

Bastos RKX, Mara DD. 1995. The bacterial quality of salad crops drip and furrow irrigated with waste stabilization pond effluent: An evaluation of the who guidelines. Water Sci. Technol. 31:425–430; doi:http://dx.doi.org/10.1016/0273-1223(95)00529-V.

Bichai F, Polo-López MI, Fernández Ibañez P. 2012. Solar disinfection of wastewater to reduce contamination of lettuce crops by Escherichia coli in reclaimed water irrigation. Water Res. 46:6040–6050; doi:http://dx.doi.org/10.1016/j.watres.2012.08.024.

Calderon-Preciado D, Matamoros V, Save R, Munoz P, Biel C, Bayona JM. 2013. Uptake of microcontaminants by crops irrigated with reclaimed water and groundwater under real field greenhouse conditions. Environ. Sci. Pollut. Res. 20:3629–3638; doi:10.1007/s11356-013-1509-0.

Carlander A, Schonning C, Stenstrom TA. 2009. Energy forest irrigated with wastewater: a comparative microbial risk assessment. J Water Heal. 7:413–433; doi:10.2166/wh.2009.020.

Castro E, Manas P, De Las Heras J. 2013. EFFECTS OF WASTEWATER IRRIGATION IN SOIL PROPERTIES AND HORTICULTURAL CROP (LACTUCA SATIVA L.). J. Plant Nutr. 36:1659–1677; doi:10.1080/01904167.2013.805221.

Choi C, Song I, Stine S, Pimentel J, Gerba C. 2004. Role of irrigation and wastewater reuse: comparison of subsurface irrigation and furrow irrigation. Water Sci. Technol. 50: 61–68.

Diallo MBC, Anceno AJ, Tawatsupa B, Houpt ER, Wangsuphachart V, Shipin O V. 2008. Infection risk assessment of diarrhea-related pathogens in a tropical canal network. Sci. Total Environ. 407:223–232; doi:http://dx.doi.org/10.1016/j.scitotenv.2008.09.034.

Dodgen LK, Li J, Parker D, Gan JJ. 2013. Uptake and accumulation of four PPCP/EDCs in two leafy vegetables. Environ. Pollut. 182:150–156; doi:10.1016/j.envpol.2013.06.038.

Downs TJ, Cifuentes-Garcia E, Suffet IM. 1999. Risk screening for exposure to groundwater pollution in a wastewater irrigation district of the Mexico City region. Environ. Health Perspect. 107:553–561; doi:10.2307/3434397.

Duan N, Wang XL, Liu XD, Lin C, Hou J. 2010. Effect of anaerobic fermentation residues on a chromium-contaminated soil-vegetable system. In *International Conference on Ecological Informatics and Ecosystem Conservation* (Z. Yang and B. Cheneds.), Vol. 2 of *Procedia Environmental Sciences*, pp. 1585–1597.

Ensink JHJ, Mahmood T, Dalsgaard A. 2007. Wastewater-irrigated vegetables: market handling versus irrigation water quality. Trop. Med. Int. Heal. 12:2–7; doi:10.1111/j.1365-3156.2007.01935.x.

Farahat E, Linderholm HW. 2013. Effects of treated wastewater irrigation on size-structure, biochemical products and mineral content of native medicinal shrubs. Ecol. Eng. 60:235–241; doi:10.1016/j.ecoleng.2013.07.050.

Fasciolo GE, Meca MI, Gabriel E, Morabito J. 2002. Effects on crops of irrigation with treated municipal wastewaters. Water Sci Technol 45: 133–138.

Ferrer A, Nguyen-Viet H, Zinsstag J. 2012. Quantification of Diarrhea Risk Related to Wastewater Contact

in Thailand. Ecohealth 9:49–59; doi:10.1007/s10393-012-0746-x.

Forslund A, Ensink JHJ, Battilani A, Kljujev I, Gola S, Raicevic V, et al. 2010. Faecal contamination and hygiene aspect associated with the use of treated wastewater and canal water for irrigation of potatoes (Solanum tuberosum). Agric. Water Manag. 98:440–450; doi:10.1016/j.agwat.2010.10.007.

Forslund A, Ensink JHJ, Markussen B, Battilani A, Psarras G, Gola S, et al. 2012. Escherichia coli contamination and health aspects of soil and tomatoes (Solanum lycopersicum L.) subsurface drip irrigated with on-site treated domestic wastewater. Water Res. 46:5917–5934; doi:http://dx.doi.org/10.1016/j.watres.2012.08.011.

Friedler E, Lahav O, Jizhaki H, Lahav T. 2006. Study of urban population attitudes towards various wastewater reuse options: Israel as a case study. J. Environ. Manage. 81:360–370; doi:10.1016/j.jenvman.2005.11.013.

Gemmell ME, Schmidt S. 2012. Microbiological assessment of river water used for the irrigation of fresh produce in a sub-urban community in Sobantu, South Africa. Food Res. Int. 47:300–305; doi:http://dx.doi.org/10.1016/j.foodres.2011.07.016.

Grangier C, Qadir M, Singh M. 2012. Health Implications for Children in Wastewater-Irrigated Peri-Urban Aleppo, Syria. Water Qual. Expo. Heal. 4:187–195; doi:10.1007/s12403-012-0078-7.

Gupta N, Khan DK, Santra SC. 2008. An assessment of heavy metal contamination in vegetables grown in wastewater-irrigated areas of Titagarh, West Bengal, India. Bull. Environ. Contam. Toxicol. 80:115–118; doi:10.1007/s00128-007-9327-z.

Gupta N, Khan DK, Santra SC. 2012. Heavy metal accumulation in vegetables grown in a long-term wastewater-irrigated agricultural land of tropical India. Environ. Monit. Assess. 184:6673–6682; doi:10.1007/s10661-011-2450-7.

Gupta N, Khan DK, Santra SC. 2009. Prevalence of intestinal helminth eggs on vegetables grown in wastewater-irrigated areas of Titagarh, West Bengal, India. Food Control 20:942–945; doi:10.1016/j.foodcont.2009.02.003.

Hamilton AJ, Stagnitti F, Premier R, Boland AM, Hale G. 2006. Quantitative microbial risk assessment models for consumption of raw vegetables irrigated with reclaimed water. Appl. Environ. Microbiol. 72:3284–3290; doi:10.1128/aem.72.5.3284-3290.2006.

Jan FA, Ishaq M, Khan S, Ihsanullah I, Ahmad I, Shakirullah M. 2010. A comparative study of human health risks via consumption of food crops grown on wastewater irrigated soil (Peshawar) and relatively clean water irrigated soil (lower Dir). J. Hazard. Mater. 179:612–621; doi:10.1016/j.jhazmat.2010.03.047.

Jang T, Lee SB, Sung CH, Lee HP, Park SW. 2010. Safe application of reclaimed water reuse for agriculture in Korea. Paddy Water Environ. 8:227–233; doi:10.1007/s10333-010-0203-9.

Kalavrouziotis IK, Robolas P, Koukoulakis PH, Papadopoulos AH. 2008. Effects of municipal reclaimed wastewater on the macro- and micro-elements status of soil and of Brassica oleracea var. Italica, and B. oleracea var. Gemmifera. Agric. Water Manag. 95:419–426; doi:http://dx.doi.org/10.1016/j.agwat.2007.11.004.

Kang MS, Kim SM, Park SW, Lee JJ, Yoo KH. 2007. Assessment of reclaimed wastewater irrigation impacts on water quality, soil, and rice cultivation in paddy fields. J. Environ. Sci. Heal. Part a-Toxic/Hazardous Subst. Environ. Eng. 42:439–445; doi:10.1080/10934520601187633.

Keraita B, Drechsel P, Konradsen F. 2008. Perceptions of farmers on health risks and risk reduction measures in wastewater-irrigated urban vegetable farming in Ghana. J. Risk Res. 11:1047–1061; doi:10.1080/13669870802380825.

Khai NM, Ha PQ, Oborn I. 2007. Nutrient flows in small-scale peri-urban vegetable farming systems in Southeast Asia - A case study in Hanoi. Agric. Ecosyst. Environ. 122:192–202;

doi:10.1016/j.agee.2007.01.003.

Khan MU, Malik RN, Muhammad S. 2013. Human health risk from Heavy metal via food crops consumption with wastewater irrigation practices in Pakistan. Chemosphere 93:2230–2238; doi:10.1016/j.chemosphere.2013.07.067.

Khan S, Aijun L, Zhang S, Hu Q, Zhu Y-G. 2008a. Accumulation of polycyclic aromatic hydrocarbons and heavy metals in lettuce grown in the soils contaminated with long-term wastewater irrigation. J. Hazard. Mater. 152:506–515; doi:http://dx.doi.org/10.1016/j.jhazmat.2007.07.014.

Khan S, Cao Q, Zheng YM, Huang YZ, Zhu YG. 2008b. Health risks of heavy metals in contaminated soils and food crops irrigated with wastewater in Beijing, China. Environ. Pollut. 152:686–692; doi:10.1016/j.envpol.2007.06.056.

Lente I, Keraita B, Drechsel P, Ofosu-Anim J, Brimah A. 2012. Risk Assessment of Heavy-Metal Contamination on Vegetables Grown in Long-Term Wastewater Irrigated Urban Farming Sites in Accra, Ghana. Water Qual. Expo. Heal. 4:179–186; doi:10.1007/s12403-012-0077-8.

Lim K-Y, Jiang SC. 2013. Reevaluation of health risk benchmark for sustainable water practice through risk analysis of rooftop-harvested rainwater. Water Res. 47:7273–7286; doi:http://dx.doi.org/10.1016/j.watres.2013.09.059.

Mahmood A, Malik RN. 2013. Human health risk assessment of heavy metals via consumption of contaminated vegetables collected from different irrigation sources in Lahore, Pakistan. Arab. J. Chem.; doi:http://dx.doi.org/10.1016/j.arabjc.2013.07.002.

Manas P, Castro E, de Las Heras J. 2009. Irrigation with treated wastewater: effects on soil, lettuce (Lactuca sativa L.) crop and dynamics of microorganisms. J Env. Sci Heal. A Tox Hazard Subst Env. Eng 44:1261–1273; doi:10.1080/10934520903140033.

Manios T, Papagrigoriou I, Daskalakis G, Sabathianakis I, Terzakis S, Maniadakis K, et al. 2006. Evaluation of primary and secondary treated and disinfected wastewater irrigation of tomato and cucumber plants under greenhouse conditions, regarding growth and safety considerations. Water Environ. Res. 78:797–804; doi:10.2175/106143006x99830.

Mapanda F, Mangwayana EN, Nyamangara J, Giller KE. 2007. Uptake of heavy metals by vegetables irrigated using wastewater and the subsequent risks in Harare, Zimbabwe. Phys. Chem. Earth, Parts A/B/C 32:1399–1405; doi:http://dx.doi.org/10.1016/j.pce.2007.07.046.

Mara DD, Sleigh PA, Blumenthal UJ, Carr RM. 2007. Health risks in wastewater irrigation: Comparing estimates from quantitative microbial risk analyses and epidemiological studies. J. Water Health 5:39–50; doi:10.2166/wh.2006.055.

Melloul AA, Hassani L, Rafouk L. 2001. Salmonella contamination of vegetables irrigated with untreated wastewater. World J. Microbiol. Biotechnol. 17:207–209; doi:10.1023/A:1016686501953.

Mendoza-Espinosa LG, Cabello-Pasini A, Macias-Carranza V, Daessle-Heuser W, Orozco-Borbon M V, Quintanilla-Montoya AL. 2008. The effect of reclaimed wastewater on the quality and growth of grapevines. Water Sci. Technol. 57:1445–1450; doi:10.2166/wst.2008.242.

Minhas PS, Sharma N, Yadav RK, Joshi PK. 2006. Prevalence and control of pathogenic contamination in some sewage irrigated vegetable, forage and cereal grain crops. Bioresour. Technol. 97:1174–1178; doi:http://dx.doi.org/10.1016/j.biortech.2005.05.006.

Mojid MA, Wyseure GCL. 2013. Implications of municipal wastewater irrigation on soil health from a study in Bangladesh. Soil Use Manag. 29:384–396; doi:10.1111/sum.12056.

Mojid MA, Wyseure GCL, Biswas SK, Hossain ABMZ. 2010. Farmers' perceptions and knowledge in using wastewater for irrigation at twelve peri-urban areas and two sugar mill areas in Bangladesh. Agric. Water Manag. 98:79–86; doi:http://dx.doi.org/10.1016/j.agwat.2010.07.015.

Moradmand M, Harchegani HB. 2011. Treated municipal wastewater irrigation effect on lead content and health risks of nickel in soil and pepper in Shahrekord, Iran. Desalin. Water Treat. 28:42–45; doi:10.5004/dwt.2011.2198.

Mukhtar M, Ensink J, Van der Hoek W, Amerasinghe FP, Konradsen F. 2006. Importance of waste stabilization ponds and wastewater irrigation in the generation of vector mosquitoes in Pakistan. J Med Entomol 43: 996–1003.

Mukhtar M, Herrel N, Amerasinghe FP, Ensink J, van der Hoek W, Konradsen F. 2003. Role of wastewater irrigation in mosquito breeding in south Punjab, Pakistan. Southeast Asian J Trop Med Public Heal. 34: 72–80.

Munoz I, Tomas N, Mas J, Garcia-Reyes JF, Molina-Diaz A, Fernandez-Alba AR. 2010. Potential chemical and microbiological risks on human health from urban wastewater reuse in agriculture. Case study of wastewater effluents in Spain. J. Environ. Sci. Heal. Part B-Pesticides Food Contam. Agric. Wastes 45:300–309; doi:10.1080/03601231003704648.

Mutengu S, Hoko Z, Makoni FS. 2007. An assessment of the public health hazard potential of wastewater reuse for crop production. A case of Bulawayo city, Zimbabwe. Phys. Chem. Earth 32:1195–1203; doi:10.1016/j.pce.2007.07.019.

Nabulo G, Young SD, Black CR. 2010. Assessing risk to human health from tropical leafy vegetables grown on contaminated urban soils. Sci. Total Environ. 408:5338–5351; doi:http://dx.doi.org/10.1016/j.scitotenv.2010.06.034.

Navarro I, Jimenez B. 2011. Evaluation of the WHO helminth eggs criteria using a QMRA approach for the safe reuse of wastewater and sludge in developing countries. Water Sci. Technol. 63:1499–1505; doi:10.2166/wst.2011.394.

Njenga M, Karanja N, Magoiya J. 2011. Risks Associated with Urban Wastewater Irrigation and Production of Traditional African Vegetable (TAVs) Seeds in Nairobi, Kenya. Acta Hortic. 911: 295–300.

Palacios MP, Lupiola P, Tejedor MT, Del-Nero E, Pardo A, Pita L. 2001. Climatic effects on Salmonella survival in plant and soil irrigated with artificially inoculated wastewater: preliminary results. Water Sci. Technol. 43: 103–108.

Palese AM, Pasquale V, Celano G, Figliuolo G, Masi S, Xiloyannis C. 2009. Irrigation of olive groves in Southern Italy with treated municipal wastewater: Effects on microbiological quality of soil and fruits. Agric. Ecosyst. Environ. 129:43–51; doi:http://dx.doi.org/10.1016/j.agee.2008.07.003.

Pandey R, Shubhashish K, Pandey J. 2012. Dietary intake of pollutant aerosols via vegetables influenced by atmospheric deposition and wastewater irrigation. Ecotoxicol. Environ. Saf. 76:200–208; doi:http://dx.doi.org/10.1016/j.ecoenv.2011.10.004.

Papadopoulos F, Parissopoulos G, Papadopoulos A, Zdragas I, Ntanos D, Prochaska C, et al. 2009. Assessment of Reclaimed Municipal Wastewater Application on Rice Cultivation. Environ. Manage. 43:135–143; doi:10.1007/s00267-008-9221-4.

Pedrero F, Alarcón JJ. 2009. Effects of treated wastewater irrigation on lemon trees. Desalination 246:631–639; doi:http://dx.doi.org/10.1016/j.desal.2008.07.017.

Pedrero F, Allende A, Gil MI, Alarcón JJ. 2012. Soil chemical properties, leaf mineral status and crop production in a lemon tree orchard irrigated with two types of wastewater. Agric. Water Manag. 109:54–60; doi:http://dx.doi.org/10.1016/j.agwat.2012.02.006.

Peters D, Ngai DD. 2004. Agro-processing waste assessment and management in peri-urban Hanoi, Vietnam. J. Sustain. Agric. 25:69–95; doi:10.1300/J064v25n01_07.

Petterson SR, Ashbolt NJ. 2001. Viral risks associated with wastewater reuse: modeling virus persistence on wastewater irrigated salad crops. Water Sci Technol 43: 23–26.

Petterson SR, Ashbolt NJ, Sharma A. 2001. Microbial risks from wastewater irrigation of salad crops: A screening-level risk assessment. Water Environ. Res. 73:667–672; doi:10.2175/106143001x143402.

Platzer M, Caceres V, Fong N. 2004. The reuse of treated wastewater for agricultural purposes in Nicaragua; Central America. Water Sci. Technol. 50: 293–300.

Qadir M, Ghafoor A, Murtaza G, MURTAZA G. 2000. Cadmium Concentration in Vegetables Grown on Urban Soils Irrigated with Untreated Municipal Sewage. Environ. Dev. Sustain. 2:13–21; doi:10.1023/A:1010061711331.

Ramadan AA, Mandil H. 2009. Wastewater Irrigation and Soil Contamination Effect on Some Leafy Vegetables Grown in Syrian Aleppo City. Asian J. Chem. 21: 3243–3252.

Rusan MJM, Hinnawi S, Rousan L. 2007. Long term effect of wastewater irrigation of forage crops on soil and plant quality parameters. Desalination 215:143–152; doi:10.1016/j.desal.2006.10.032.

Rutkowski T, Raschid-Sally L, Buechler S. 2007. Wastewater irrigation in the developing world - Two case studies from the Kathmandu Valley in Nepal. Agric. Water Manag. 88:83–91; doi:10.1016/j.agwat.2006.08.012.

Sharma RK, Agrawal M, Marshall F. 2007. Heavy metal contamination of soil and vegetables in suburban areas of Varanasi, India. Ecotoxicol. Environ. Saf. 66:258–266; doi:10.1016/j.ecoenv.2005.11.007.

Shuval H, Lampert Y, Fattal B. 1997. Development of a risk assessment approach for evaluating wastewater reuse standards for agriculture. Water Sci. Technol. 35:15–20; doi:10.1016/s0273-1223(97)00228-x.

Silverman AI, Akrong MO, Amoah P, Drechsel P, Nelson KL. 2013. Quantification of human norovirus GII, human adenovirus, and fecal indicator organisms in wastewater used for irrigation in Accra, Ghana. J. Water Health 11:473–488; doi:10.2166/wh.2013.025.

Singh A, Sharma RK, Agrawal M, Marshall FM. 2010. Health risk assessment of heavy metals via dietary intake of foodstuffs from the wastewater irrigated site of a dry tropical area of India. Food Chem. Toxicol. 48:611–619; doi:http://dx.doi.org/10.1016/j.fct.2009.11.041.

Srinivasan JT, Reddy VR. 2009. Impact of irrigation water quality on human health: A case study in India. Ecol. Econ. 68:2800–2807; doi:http://dx.doi.org/10.1016/j.ecolecon.2009.04.019.

Tai YP, Lu HP, Li ZA, Zhuang P, Zou B, Xia HP, et al. 2013. Purification of contaminated paddy fields by clean water irrigation over two decades. Environ. Geochem. Health 35:657–666; doi:10.1007/s10653-013-9548-5.

Tang JC, Bai XR, Zhang WJ. 2011. Cadmium Pollution and its Transfer in Agricultural Systems in the Suburbs of Tianjin, China. Soil Sediment Contam. 20:722–732; doi:10.1080/15320383.2011.594112.

Wang C, Chen Y, Liu J, Wang J, Li X, Zhang Y, et al. 2013a. Health risks of thallium in contaminated arable soils and food crops irrigated with wastewater from a sulfuric acid plant in western Guangdong province, China. Ecotoxicol Env. Saf 90:76–81; doi:10.1016/j.ecoenv.2012.12.014.

Wang N, Li HB, Long JL, Cai C, Dai JL, Zhang J, et al. 2012a. Contamination, source, and input route of polycyclic aromatic hydrocarbons in historic wastewater-irrigated agricultural soils. J. Environ. Monit. 14:3076–3085; doi:10.1039/c2em30650f.

Wang QR, Cui YS, Liu XM, Dong YT, Christie P. 2003. Soil contamination and plant uptake of heavy metals at polluted sites in China. J. Environ. Sci. Heal. Part a-Toxic/Hazardous Subst. Environ. Eng. 38:823–838; doi:10.1081/ese-120018594.

Wang X, Ding WG, Nan ZR, Liao Q, Wu WF. 2013b. Fraction of Cd in oasis soil and its bioavailability to commonly grown crops in Northwest China. Environ. Earth Sci. 70:471–479; doi:10.1007/s12665-013-2374-3.

Wang X, Nan ZR, Ding WG, Wang SL, Wang ZW, Yang YM, et al. 2012b. Chemical Fraction of Heavy Metals in an Oasis Soil and Their Bioavailability to Cole Crops. Arid L. Res. Manag. 26:166–180;

doi:10.1080/15324982.2012.656177.

Wang YC, Qiao M, Liu YX, Zhu YG. 2012c. Health risk assessment of heavy metals in soils and vegetables from wastewater irrigated area, Beijing-Tianjin city cluster, China. J. Environ. Sci. 24:690–698; doi:10.1016/s1001-0742(11)60833-4.

Weldesilassie AB, Boelee E, Drechsel P, Dabbert S. 2011. Wastewater use in crop production in peri-urban areas of Addis Ababa: impacts on health in farm households. Environ. Dev. Econ. 16:25–49; doi:10.1017/s1355770x1000029x.

Wu X, Ernst F, Conkle JL, Gan J. 2013. Comparative uptake and translocation of pharmaceutical and personal care products (PPCPs) by common vegetables. Env. Int 60:15–22; doi:10.1016/j.envint.2013.07.015.

Yang QW, Lan CY, Wang HB, Zhuang P, Shu WS. 2006. Cadmium in soil–rice system and health risk associated with the use of untreated mining wastewater for irrigation in Lechang, China. Agric. Water Manag. 84:147–152; doi:http://dx.doi.org/10.1016/j.agwat.2006.01.005.

Zhang J, Yang JC, Wang RQ, Hou H, Du XM, Fan SK, et al. 2013. Effects of pollution sources and soil properties on distribution of polycyclic aromatic hydrocarbons and risk assessment. Sci. Total Environ. 463:1–10; doi:10.1016/j.scitotenv.2013.05.066.
